# Upregulated expression of HOXB7 in intrahepatic cholangiocarcinoma is associated with tumor cell metastasis and poor prognosis

**DOI:** 10.1038/s41374-018-0150-4

**Published:** 2019-01-21

**Authors:** Longfei Dai, Wendi Hu, Zhenjie Yang, Diyu Chen, Bin He, Yunhao Chen, Lin Zhou, Haiyang Xie, Jian Wu, Shusen Zheng

**Affiliations:** 1 Division of Hepatobiliary and Pancreatic Surgery, Department of Surgery, First Affiliated Hospital, School of Medicine, Zhejiang University, Hangzhou, 310000 China; 2 Key Laboratory of Combined Multi-organ Transplantation, Ministry of Public Health, Hangzhou, 310000 China; 3 Key Laboratory of Organ Transplantation, Zhejiang Province, Hangzhou, 310003 China; 4 Collaborative Innovation Center for Diagnosis Treatment of Infectious Diseases, Hangzhou, 310000 China

**Keywords:** Tumour biomarkers, Metastasis

## Abstract

Homeobox B7 (HOXB7) protein is reported to be aberrantly expressed in a variety of cancers and to play an important role in multiple cellular processes. However, the specific mechanism by which HOXB7 promotes the malignant progression of intrahepatic cholangiocarcinoma (ICC) remains unclear. Therefore, we used quantitative real-time polymerase chain reaction (PCR) to detect the expression level of HOXB7 in 38 paired ICC tissue samples. Additionally, to assess correlation between HOXB7 and ICC prognosis, we performed immunohistochemistry (IHC) using 122 ICC tissues to detect HOXB7 expression. Cell Counting Kit-8 (CCK-8) and colony formation assays were employed to assess ICC cell proliferation, and Transwell assays were performed to estimate the invasion and migration abilities of ICC cells. The capillary tube formation assay was applied to explore the angiogenic effects of HOXB7. A xenograft tumor model was established in nude mice to assess the role of HOXB7 in tumor growth and lung metastasis. The results showed higher expression of HOXB7 in ICC tissues than in noncancerous tissues, and this increased expression was significantly associated with a poor prognosis. In addition, HOXB7 overexpression enhanced capillary tube formation, invasion and migration of ICC cells in vitro, whereas HOXB7 knockdown produced the opposite results in vitro. Moreover, the role of HOXB7 in promoting tumor growth and metastasis was verified in vivo. Further investigation revealed that the expression levels of MMP2, MMP9, VEGFa, and IL8 were elevated by HOXB7 and that the ERK pathway was activated. Our results demonstrate the prognostic value of HOXB7 and its role in metastasis and angiogenesis in ICC. HOXB7 upregulated MMP2, MMP9, VEGFa, and IL8 expression via the ERK pathway to accelerate the malignant progression of ICC.

## Introduction

Cholangiocarcinoma (CCA) is an epithelial cell malignancy that can originate in different locations of the biliary tree [1] and the second most common primary hepatic neoplasm after hepatocellular carcinoma [2]. The current classification of CCA is based on anatomical location and includes intrahepatic, perihilar, and distal sites. Among these, intrahepatic cholangiocarcinoma (ICC) comprises 8–10% of CCAs and 10–20% of all primary liver tumors. Although ICC is relatively rare, global epidemiology data show that the incidence of ICC is increasing [3–5]. Moreover, CCA is one of the most fatal cancers, as its 5-year survival rates are only 30–40% after complete resection and its median survival for patients with unresectable disease is only 12–15 months [6]. However, one confounding phenomenon that clinicians experience is that none of the known specific risk factors for CCA can be detected in ICC patients. Thus, an adequate understanding of the molecular mechanisms involved in ICC progression may help us to improve the prognosis of ICC patients.

Homeobox (Hox) genes regulate cell differentiation and morphogenesis during embryonic development. These genes are characterized by the presence of a homeodomain, a signature DNA sequence that encodes 61 amino acids [7]. Hox proteins can function as monomers or homodimers to directly drive transcription of downstream targets [8]. Homeobox B7 (HOXB7) has been reported to be aberrantly expressed in a variety of cancers, including melanoma [9], breast cancer [10–15], gastric cancer [16, 17], liver cancer [18, 19], colorectal cancer [20] and esophageal cancer [21]. In addition, its expression has been correlated with clinical progression and poor outcome and may serve as a potential prognostic factor and therapeutic target [11, 14, 19, 20, 22, 23]. HOXB7 is involved in many of the major cellular processes that occur in cancer, including proliferation, invasion, migration, angiogenesis and the epithelial–mesenchymal transition (EMT) [24]. Furthermore, HOXB7 has been reported to facilitate the migration of breast [25] and liver [18] cancer cells by inducing EMT, and ectopic expression of HOXB7 promotes angiogenesis [9, 26] and cell proliferation [17, 18]. However, some researchers have reported that HOXB7 overexpression had no effect on cellular morphology and growth rate in breast cancer cells [27]. Although the biological functions of HOXB7 in various cancers have been described, its function in the controlling tumorigenesis and tumor progression of ICC has not been well characterized.

In this study, we confirmed that HOXB7 is upregulated in ICC tissues, which is associated with poor outcomes in ICC patients. Additionally, we investigated the biological function of the HOXB7 protein in ICC cell lines. Our results suggest that overexpression of HOXB7 promotes ICC metastasis by increasing MMP2 and MMP9 expression both in vitro and in vivo. Furthermore, overexpression of HOXB7 promotes ICC angiogenesis by increasing IL8 and VEGF expression in vitro and in vivo. In contrast, HOXB7 knockdown produced the opposite results in vitro. Surprisingly, HOXB7 had no effect on ICC proliferation in vitro but could promote ICC proliferation in vivo. In summary, our study highlights the prognostic value of HOXB7 in ICC. To the best of our knowledge, this is the first report to indicate the role of HOXB7 in metastasis and angiogenesis in primary tumor samples from ICC patients and to investigate the mechanism by which HOXB7 contributes to ICC cell migration, invasion and angiogenesis.

## Materials and methods

### Clinical specimen collection

Samples from 122 patients with ICC who underwent hepatic resection between 2007 and 2013 at First Affiliated Hospital of Zhejiang University (Zhejiang, China) were obtained. This study was approved by First Affiliated Hospital of Zhejiang University Ethics Committee, and informed consent was obtained from each patient following institutional review board protocols. The histological grade of tumor differentiation was determined according to the classification proposed by Edmondson and Steiner. Overall survival (OS) was defined as the interval between the date of surgery and date of death.

### Cell culture

Human ICC cell lines (CCLP-1, HUCCT-1, HIBEC, RBE) were purchased from the Cell Bank of Type Culture Collection at the Chinese Academy of Sciences and cultured in RPMI1640 complete medium supplemented with 10% fetal bovine serum (Gibco, USA) and penicillin/streptomycin. Human umbilical vein endothelial cells (HUVECs) were obtained from AllCells (Shanghai, China; allcells.biomart.cn) and maintained in DMEM supplemented with 10% fetal bovine serum. All the cells were cultured at 37 °C in 5% CO_2_ and passaged every 3 or 4 days when they reached 80–90% confluency.

### Lentivirus production and cell transduction

Lentivirus ectopically expressing HOXB7 and a matching negative control lentivirus expressing GFP were purchased from GeneChem (Shanghai, China). Transfection processes were conducted according to the instructions provided by the manufacturer. The transduced cells were then selected in culture medium containing puromycin (1.5 μg/ml).

For siRNA transfection, cells were seeded on 6-well tissue culture plates at 40% confluence. The next day, the cells were transfected with 200 nM pooled siRNA targeting HOXB7 or IL8 or control siRNA using Lipofectamine 2000 reagent (Invitrogen) and Opti-MEM (Thermo Fisher, Waltham, MA, USA) according to the manufacturer’s instructions. siHOXB7 for HOXB7 knockdown and siNC as a negative control were purchased from Invitrogen (HSS104954, sequence 5′UCGAGCCGAGUUCCUUCAACAUGCA3′; HSS104955, sequence 5′UCUGCCUCACGGAAAGACAGAUCAA3′ and HSS179336, sequence 5′ GCGGCCGAGAGUAACUUCCGGAUCU 3′). siIL8 for IL8 knockdown and siNC as a negative control were purchased from Guangzhou RiboBio Co. (siB09112383133, sequence 5′ GCGCCAACACAGAAATTAT 3′; siB09112383145, sequence 5′CAAAGAACTGAGAGTGATT 3′).

### Histology and immunohistochemistry

Paraffin-embedded tissue samples from ICC patients were sliced into 4-μm-thick sections. Tumor tissues from mice were also sectioned at a 4-μm thickness using a thin semiautomatic microtome. All sections were deparaffinized in xylene and rehydrated in a series of graded alcohol dilutions. Antigen retrieval was performed by heating in a microwave oven. Then, the sections were incubated with 3% H_2_O_2_ for 10 min followed by 10% normal goat serum for 15 min at room temperature to block endogenous peroxidases and non-specific antigens. Histological sections were immunostained overnight at 4 °C using the following primary antibodies: anti-HOXB7 antibody (1:100, #H00003217-M03, Abnova Corporation, Taiwan), anti-CD31 antibody (1:200; GB1306; Servicebio, China) and anti-CD34 antibody (1:1000; GB13013; Servicebio, China). After rinsing with PBS three times, the sections were incubated with horseradish peroxidase (HRP)-labeled secondary antibody for 30 min at room temperature. Finally, 3,3-diaminobenzidine (DAB) was used to visualize signal development, and then the sections were counterstained with haematoxylin.

Two independent investigators on our team blinded to the pathological data used the software ImagePro Plus 6.0 software to assess the immunoreactive density. The paraffin sections were scored semiquantitatively as follows: Grade 0: 0% immunoreactive cells; Grade 1:≤10% immunoreactive cells; Grade 2:>10–50% immunoreactive cells; and Grade 3:≥50 immunoreactive cells. For statistical purposes, cases with Grade 0 and 1 were classified as low expression, whereas those with Grades 2 and 3 were classified as high expression.

### Microvessel density count

To quantify microvessel density (MVD), hotspots on tumor biopsies (areas containing the highest number of blood vessels) were identified in low-power magnification fields (×40). Then, the MVD was assessed by counting the vessel numbers in three different fields under high-power magnification (×400). An average was calculated for each case and statistically presented as the mean ± SD. The isolated immunoreactive endothelial cells or groups of endothelial cells separated by adjacent microvessels were considered quantifiable individual vessels. Visible lumens or the presence of associated red cells were not obligatory.

### Western blot analysis

After incubation, cells were lysed and sonicated to obtain a soluble protein lysate. The protein concentration was determined by the Bradford assay (Bio-Rad). Equal amounts of protein were loaded onto a 10% NuPAGE Bis-Tris Gel (Invitrogen) and electrophoresed. Then, the lysates were transferred onto PVDF membrane for 60–90 min based on the molecular weight of the target protein. After the membranes were blocked with 5% non-fat milk, they were incubated overnight at 4 °C with the following primary antibodies: anti-HOXB7 (1:200; ab51237; Abcam, USA), anti-FLAG (1:1000; SAB4200071; Sigma-Aldrich), anti-IL8 (1:1000; AP8612B; ABGENT, USA), anti-MMP2 (1:1000; #10373–2-AP; ProteinTech, USA), anti-MMP9 (1:1000; #10375-2-AP; ProteinTech, USA), VEGFa (1:1000, ab46154, Abcam, USA), anti-p-MEK1/2 (1:1000, #9154, Cell Signaling Technology), anti-p-ERK1/2 (1:2000, #4370, Cell Signaling Technology), anti-ERK (1:1000, ab17942, Abcam, USA), anti-cyclin B1 (1:1000, #12231, Cell Signaling Technology), anti-CDK4 (1:1000, #12790, Cell Signaling Technology) or anti-GAPDH (1:5000; #5174; Cell Signaling Technology). After the membranes were washed three times with Tris-buffered saline containing 0.05% Tween-20 for 10 min per wash, signals were detected with the SuperSignal West Pico Chemiluminescent Substrate (Pierce) and quantified using ImageJ 1.44 software from Wayne Rasband (National Institutes of Health, Bethesda, MD, USA). GAPDH was used as a loading control.

### RNA extraction and quantitative real-time polymerase chain reaction (qRT-PCR)

Total RNA was isolated from clinical samples using TRIzol reagent (Invitrogen) according to the manufacturer’s recommended protocol. For quantitative real-time RT–PCR analysis, a Roche LightCycler was used with the Takara SYBR Premix ExTaq system. Primers were synthesized by Shanghai Sangon Biological Engineering Technology Services Co., Ltd. The nucleotide sequences of the primers were as follows: GAPDH, 5′-AAGGTGAAGGTCGGAGTCAA-3′ and 5′-AATGAAGGGGTCATTGATGG-3′; and HOXB7, 5′-ATCTACCCCTGGATGCGAAGCT-3′ and 5′-GCGTCAGGTAGCGATTGTAGTG-3′. Each sample was assessed in triplicate. Gene expression in the tumor cell lines or clinical samples was calculated relative to the GAPDH expression using the 2^−ΔΔCt^ method.

### Cell viability and colony formation assays

Cell viability was detected with Cell Counting Kit-8 (CCK-8) (DOJINDO Laboratories, Kuma-moto, Japan). ICC cells were seeded in 96-well plates at 1200 cells/well and incubated in humidified incubator for 24, 48, 72, or 96 h. After the supernatant removed, 90 μl of medium and 10 μl of CCK-8 solution were added to every well, and the plates were incubated for 1 h. The absorbance of each well was detected at 450 nm using a microplate reader (BioTek, USA) according to the manufacturer’s instructions.

For the colony formation assay, cells were seeded into six-well plates at a concentration of 2000 cells/well and cultured at 37 °C for 10–16 days based on the growth characteristics of each cell line. Then, the cells were fixed with 100% methanol and stained with 0.1% crystal violet. Cell colonies were counted macroscopically by ImagePro Plus 5.0 (Media Cybernetics).

### Cell cycle analysis

After stable ICC cell lines overexpressing HOXB7 were established, an equal number of transduced or control cells was seeded into culture dishes and then incubated for 24 h. Next, the medium was replaced with serum-free medium. After incubation for another 24 h, the cells (1 × 10^6^) were fixed in 75% ethanol at −20 °C for 24 h. The fixed cells were stained according to the protocol for the Cycle TEST™ PLUS DNA Reagent Kit (BD Biosciences) and analyzed by flow cytometry (Beckman Coulter FC 500).

### Migration and invasion assays

Migration and invasion assays were performed in the BD Falcon 24-well insert system (BD Biosciences, San Jose, CA) according to the manufacturer’s instruction. For the migration assays, cells in serum-free medium were seeded on the upper chamber with a non-coated membrane. For the invasion assays, filters were precoated for 2 h with 35 μl of solution comprising Matrigel (BD Biosciences, USA) and RPMI 1640 at a 1:7 ratio. For both experiments, 5 × 10^4^ ICC cells were seeded into the upper chamber, and culture medium containing 10% FBS was added to the lower chamber. The cells were incubated for 24 h (migration assay) and 48 h (invasion assay). Non-migratory or non-invasive cells were removed from the upper surface of the filter. Cells on the lower surface of the membrane were stained by using a Wright-Giemsa Stain Kit (Nanjing Jiancheng Bioengineering Institute, China). Cell numbers were counted under an optical microscope. Each experiment was repeated at least three times.

### Tumor cell conditioned medium (TCM) and capillary tube formation assay

After stable ICC cell lines overexpressing HOXB7 were established, an equal number of transduced or control cells was seeded into culture dishes and then incubated for 24 h. Next, the medium was replaced with serum-free medium. After incubation for another 24 h, the medium was collected, centrifuged for 10 min at 5000 × *g* and stored at −80 °C until further use.

For the capillary tube formation assay, HUVECs were seeded in Matrigel-coated 96-well plates in 75% TCM at a density of 2 × 10^4^ cells/well. After the cells were incubated for 6–8 h, the capillary-like structures of the HUVECs were photographed under an inverted microscope. The branch points of the formed tubes, which represent the degree of angiogenesis in vitro, were scanned and quantitated at ×100 magnification.

### Tumor growth and metastasis model in nude mice

ICC cells (2 × 10^6^ cells per mouse for both CCLP-1 and HUCCT-1) were resuspended in 100 μl of PBS and subcutaneously injected into the left flank of the mice. Tumor volume was calculated according to the following formula: larger diameter × (smaller diameter)^2^/2. On day 30 after injection, the subcutaneous tumors were removed for weighing and immunostaining. To evaluate the effect of HOXB7 on the metastatic ability of ICC cells, we established a metastasis model. ICC cells were resuspended in 100 μl of PBS and injected into the tail vein of nude mice (1 × 10^6^ cells per mouse for CCLP-1). After 8 weeks, the mice were sacrificed to harvest the lung tissues. H&E staining was performed to analyze the tumor clusters in the lung tissues. All the animal experiments met the requirements of the guidelines of the National Institutes of Health (Guide for the Care and Use of Laboratory Animals, 2011).

### Statistical analysis

All statistical analyses were performed using SPSS 17.0 software (SPSS). Differences between two groups were examined using a two-tailed paired Student’s *t*-test. The chi-square test was used to evaluate any potential association between HOXB7 expression and the clinicopathological parameters. Survival data were used to establish Kaplan–Meier curves, and the differences among the groups were analyzed by the log-rank test. *P* values ≤ 0.05 were considered statistically significant.

## Results

### HOXB7 expression is upregulated in human ICC tissues and correlates with poor prognosis of ICC

**Fig. 1:**
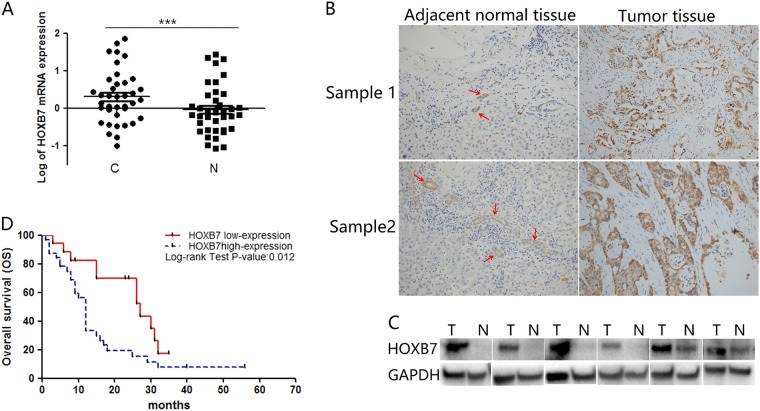
HOXB7 is highly expressed in ICC tissues and predicts poor prognosis of patients. **a** Log_10_ values of quantitative real-time PCR analysis of HOXB7 expression in 38 paired ICC and adjacent noncancerous tissues from human patients. ***P < 0.001. **b** Immunohistochemical (IHC) staining of HOXB7 expression in 122 paired ICC and paratumor tissues from human patients. Representative views are shown. The red arrow indicates the normal bile duct. **c** HOXB7 protein expression in ICC (T) and normal (N) tissues. **d** Overall survival time after surgery of 49 patients with ICC categorized as “HOXB7 low” or “HOXB7 high” was compared. *P* < 0.05

To examine the correlation between HOXB7 and ICC prognosis, paired tumor tissues and adjacent noncancerous tissues from 38 patients were examined by quantitative real-time PCR. As shown in Fig. 1a, the log_10_ value of HOXB7 mRNA expression was significantly higher in the ICC tissues than in the adjacent tissues (*P* < 0.001). Due to the heterogeneity between different samples, HOXB7 expression varied in both tumor and non-tumor tissues. This variation can also be attributed to the presence of interstitial tissue. However, HOXB7 expression was higher in ICC tissues than in adjacent tissues for most patients (73.4%, 28 of 38 patients). To confirm the qRT-PCR results, we measured HOXB7 expression in 122 paired ICC and noncancerous tissues by IHC, which revealed that HOXB7 was upregulated in ICC tumors compared with adjacent bile duct tissues (Fig. 1b). In agreement with the PCR results, western blotting showed that HOXB7 protein expression was upregulated in ICC (Fig. 1c).

To determine the impact of high levels of HOXB7 expression on the prognosis of ICC patients, we analyzed differences in OS among 49 ICC patients for whom prognostic statistics were available. As shown in Fig. 1d, subjects with low HOXB7 expression (*n* = 17) had a median survival time of 27 months. In sharp contrast, subjects with high HOXB7 expression (*n* = 32) had a median survival time of 12.0 months. These data indicate that HOXB7 is associated with a significant reduction in survival. In summary, our data confirmed that aberrant overexpression of HOXB7 in ICC is significantly correlated with poor prognosis.

### HOXB7 has no effect on proliferation in vitro but promotes ICC tumorigenesis in vivo

**Fig. 2:**
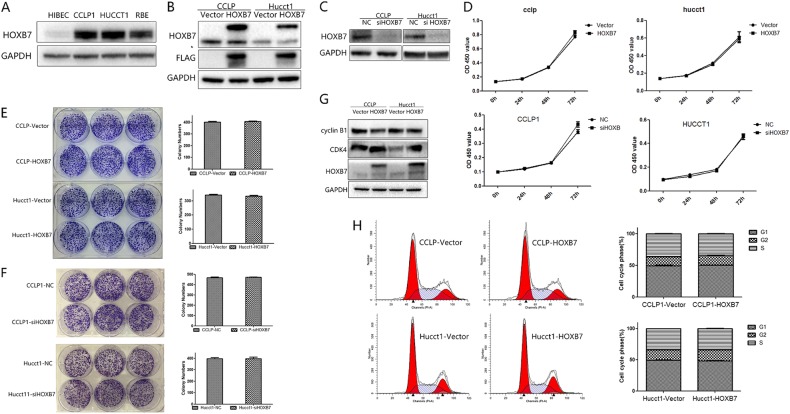
HOXB7 has no effect on ICC cell proliferation in vitro. **a** HOXB7 protein expression in ICC cell lines and a normal bile duct cell line (HIBEC). **b** CCLP-1 and HUCCT-1 cells were infected with lentivirus containing recombinant HOXB7, the expression of which was measured by western blotting. GAPDH was used as a loading control. **c** The protein levels in HOXB7 knockdown ICC cell lines were measured by western blotting. GAPDH was used as a control. **d** The proliferative ability of cells in vitro after infection was evaluated by the Cell Counting Kit-8 (CCK-8) assay. Data are shown as the mean (*n* = 3) ± SD; experiments were performed in triplicate. **e**, **f** Representative images of the colony formation assay in CCLP-1 and HUCCT-1 cells. Quantification of colony number. Data are shown as the mean (*n* = 3) ± SD. NS: no significance. **g** The expression levels of CDK4 and cyclin B1 in ICC cells were analyzed by Western blotting. **h** The cell cycle distribution of CCLP-1 and HUCCT-1 cells overexpressing HOXB7 did not obviously differ. Experiments were performed in triplicate

After verifying the association between HOXB7 and ICC prognosis, we then attempted to identify the biological function of HOXB7 with regard to ICC cell growth. First, we compared HOXB7 expression in ICC cell lines and a normal bile duct cell line (HIBEC). As shown in Fig. 2a, higher levels of HOXB7 were observed in ICC cell lines. Because CCLP-1 and HUCCT-1 are prone to tumorigenesis, we chose these two cell lines for functional experiments. We transduced CCLP-1 and HUCCT-1 cells with lentivirus carrying HOXB7 to stably overexpress HOXB7. The levels of HOXB7 and ectopic FLAG in CCLP-1 and HUCCT-1 cells were validated (Fig. 2b). However, the CCK-8 assay showed that HOXB7 overexpression did not increase the proliferation of either CCLP-1 and HUCCT-1 cells compared with that in the corresponding control cells (Fig. 2d). The colony formation assay revealed that HOXB7-overexpressing cells formed colonies in a similar manner as control cells (Fig. 2e). To better understand the biological function of HOXB7, we knocked down HOXB7 expression in ICC cells (Fig. 2c). As shown in Fig. 2d, f, HOXB7 had no effect on proliferation in vitro. Moreover, cell cycle analysis showed that HOXB7 overexpression did not remarkably lead to cell cycle progression in CCLP-1 and HUCCT-1 cells (Fig. 2h). Then, we detected the cell cycle markers cyclin dependent kinase 4 (CDK4) and cyclin B1 (Fig. 2g). CDK4 plays a specific role in tumorigenesis and development, which can expedite cell proliferation [28, 29]. Cyclin B1 activation was found to promote cell proliferation and stimulate the replication of cells [30, 31]. However, the divergent effects on CDK4 and cyclin B1 protein levels, which increased and decreased, respectively, are difficult to explain. This conflicting result may be the ultimate reason that no change in the cell cycle was observed.

**Fig. 3:**
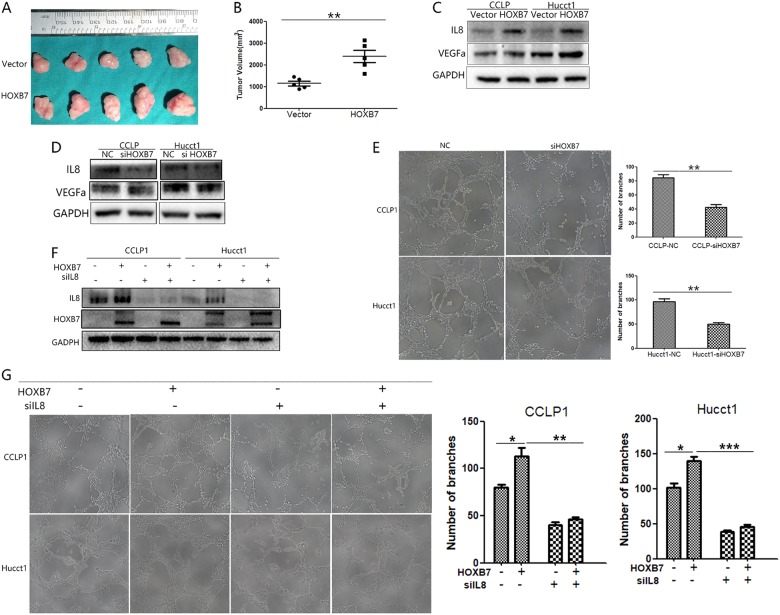
HOXB7 overexpression promotes CCLP-1 cell tumorigenicity in vivo and angiogenesis in vitro. **a** Tumors dissected from nude mice injected with cells expressing either Vector or HOXB7. **b** The tumor volume was calculated with calipres just after mice were sacrificed. Data are shown as the mean (*n* = 5) ± SD, **P < 0.01. **c**, **d** Expression of VEGFa and IL8 in ICC cells was analyzed by western blotting. **e** Capillary tube formation assays were used to detect the angiogenic effects. Data are represented as the mean ± SEM of triplicate experiments. **P < 0.01. **f** Protein levels in HOXB7-overexpressing ICC cell lines with IL8 knockdown were measured by western blotting. GAPDH was used as a control. **g** Capillary tube formation assays were used to detect angiogenic effects. Data are presented as the mean ± SEM of triplicate experiments. *P < 0.05, **P < 0.01,***P < 0.001

**Fig. 4:**
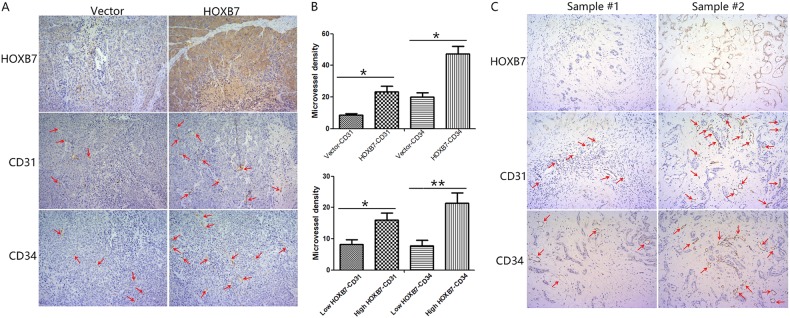
HOXB7 could promote angiogenesis in vivo. **a** IHC assays were performed to explore the protein levels of HOXB7, CD31, and CD34 in subcutaneous xenograft tumors. Representative images are shown at ×200magnification. **b** MVD was evaluated based on CD31 and CD34 staining. Data are shown as the mean ± SD, *P < 0.05, **P < 0.01. **c** IHC assays were performed to explore the protein levels of HOXB7, CD31, and CD34 in ICC tumors. Representative images are shown at ×200magnification

HOXB7 has been reported to promote cancer cell proliferation [17, 18], but other researchers have stated that this protein is not involved in proliferation [27]. As its effect on proliferation is disputable in other cancers, we established a xenograft tumor model in vivo to elucidate its true effects. Interestingly, larger tumors formed in mice injected with HOXB7-overexpressing CCLP-1 cells (Fig. 3a). Further assessment revealed that HOXB7 expression promoted tumor growth and resulted in larger tumors (Fig. 3b, *P* < 0.05). In addition to the gross observations of differences in tumor volume, IHC staining showed that tumors formed by HOXB7-overpressing cells have stronger HOXB7 staining than tumors formed by control cells, which indicated the successful establishment of our xenograft tumor model (Fig. 4a). In summary, HOXB7 has no effect on proliferation in vitro but can promote ICC tumorigenesis in vivo.

### HOXB7 increases angiogenesis in vitro

One major difference between in vitro and in vivo studies of cancer is that angiogenesis is necessary for tumor growth and progression in vivo. We speculate that angiogenesis may explain this discrepancy. To address this, we performed an in vitro capillary tube formation assay in CCLP-1 and HUCCT-1 cells and found that HUVECs incubated with tumor cell conditioned medium (TCM) from HOXB7-overexpressing cells displayed a greater ability to promote capillary-like structure formation than the control group (Fig. 3g). By contrast, HUVECs incubated with TCM from HOXB7 knockdown cells exhibited a decrease in capillary-like structure formation (Fig. 3e). We then detected the classic pro-angiogenic factor VEGFa by Western blotting. In line with our expectations, HOXB7 increased the protein expression of VEGFa (Fig. 3c). IL8 has been reported as a pro-angiogenic factor [32, 33] and IL8 was also upregulated when HOXB7 was overexpressed (Fig. 3c). By contrast, knockdown of HOXB7 down-regulated VEGFa and IL8 (Fig. 3d). To further clarify the function of IL8, we knocked down IL8 in HOXB7-overexpressing ICC cells (Fig. 3f). As shown in Fig. 3g, knockdown of IL8 reduced the increase in angiogenesis of ICC cells induced by HOXB7 overexpression. The above results indicate that IL8 is an important influence on angiogenesis of ICC cells.

Next, we excised the subcutaneous tumors and performed IHC to detect the expression levels of CD31and CD34, which indicates the presence of microvessels, in each group. As shown in Fig. 4a, tumors from mice injected with cells overexpressing HOXB7 displayed dramatically higher levels of CD31 and CD34 compared to tumors from cells transduced with empty vector. Further assessments showed that tumors formed from HOXB7-overexpressing cells had a higher microvessel density (MVD) than tumors from the control group (Fig. 4b). To determine the impact of high levels of HOXB7 expression on angiogenesis, we compared CD31 and CD34 expression by immunohistochemistry between HOXB7 high expression and low expression ICC tumors. As shown in Fig. 4c, ICC tumors that expressed high levels of HOXB7 displayed higher levels of CD31 and CD34. Further assessments showed that ICC tumors that expressed high levels of HOXB7 had a higher microvessel density (MVD) (Fig. 4b, Supplementary Table 1). The above results indicate that HOXB7 overexpression promotes angiogenesis, which in turn accelerates tumorigenicity in vivo.

### HOXB7 enhances the migration and invasion of ICC cells in vitro and facilitates metastasis of ICC cells in vivo

**Fig. 5:**
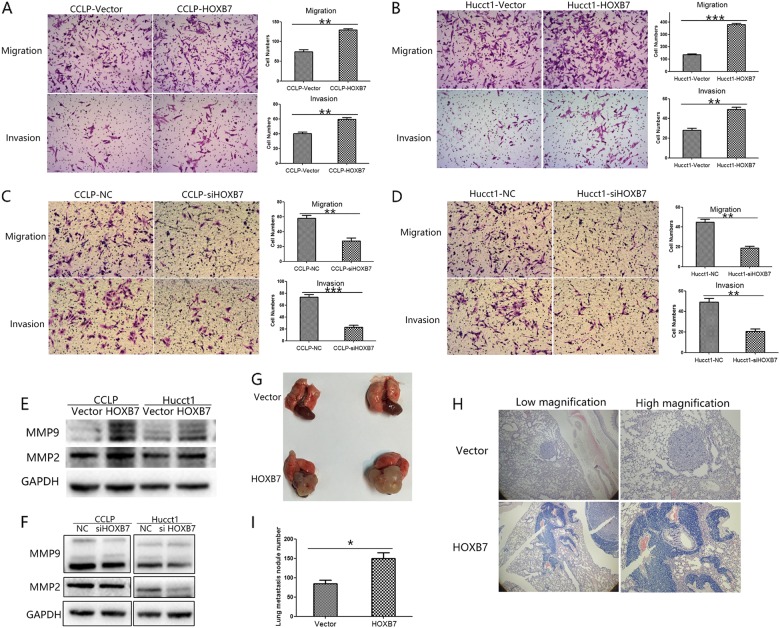
HOXB7 could promote ICC metastasis both in vitro and in vivo. **a**, **b** Migration and invasion of CCLP-1 and HUCCT-1 cells that overexpressed HOXB7 were measured by a Transwell assay. The results were quantitated by counting the migratory and invasive cells in five randomly chosen high-power fields for each replicate. **P < 0.01, ***P < 0.001. **c**, **d** The migration and invasion of CCLP-1 and HUCCT-1 cells in which HOXB7 was knocked down were measured by a Transwell assay. The results were quantitated by counting the migratory and invasive cells in five randomly chosen high-power fields for each replicate. **P < 0.01, ***P < 0.001. **e**, **f** Effects of HOXB7 overexpression on MMP-2 and MMP-9 expression were measured by Western blotting. **g** Representative image of pulmonary metastatic nodule from mice injected via tail vein with CCLP-1 cells expressing either vector or HOXB7. **h** Different magnification of pulmonary metastatic nodule from mice injected via tail vein with CCLP-1 cells expressing either vector or HOXB7; H&E staining. **i** The number of pulmonary metastasis nodules from the CCLP-1-Vector and CCLP-1-HOXB7 groups. *P < 0.05

Metastasis is also an important contributor to tumor progression, and distant metastasis can seriously affect patient prognosis. As it has been reported HOXB7 can regulate the invasion and migration of cancer cells, we examined the effects of HOXB7 overexpression on the invasion and migration abilities of ICC cells. The results of the Transwell assays showed that HOXB7 overexpression enhanced the invasion and migration abilities of CCLP-1 and HUCCT-1 cells (*P* < 0.05 for both; Fig. 5a, b). Opposite results were observed for HOXB7 knockdown cells (Fig. 5c, d). To further validate the relationship between HOXB7 and ICC metastasis, we detected the protein levels of MMP2 and MMP9 and found that HOXB7 increased the expression of both these proteins (Fig. 5e). Conversely, knockdown of HOXB7 down-regulated MMP2 and MMP9 (Fig. 5f). IL8 has been regarded as an important factor regulating cancer metastasis [34, 35] and we also observed that HOXB7 altered IL8 expression (Fig. 3c, d).

To further clarify whether HOXB7 is associated with ICC metastasis, we established a metastasis model in nude mice by injecting cancer cells into the tail vein. Overexpression of HOXB7 in CCLP-1 cells promoted metastasis and colonization of these cells in the lung (Fig. 5g). H&E staining of metastatic tissues demonstrated the colonization of ICC cells in the lung (Fig. 5h). Statistical analysis revealed that increased HOXB7 expression resulted in significantly greater numbers of macroscopic lung metastatic cancer nodules in the mouse lung tissues (HOXB7 group, 149.5 ± 31.1; Vector group, 84.75 ± 17.6; Fig. 5i, *P* < 0.05). These results indicate that elevated HOXB7 expression can promote ICC lung metastases in vivo.

### HOXB7 activates ERK1/2 signaling, and administration of an ERK1/2 inhibitor ameliorates the effects of HOXB7 in ICC cells

**Fig. 6:**
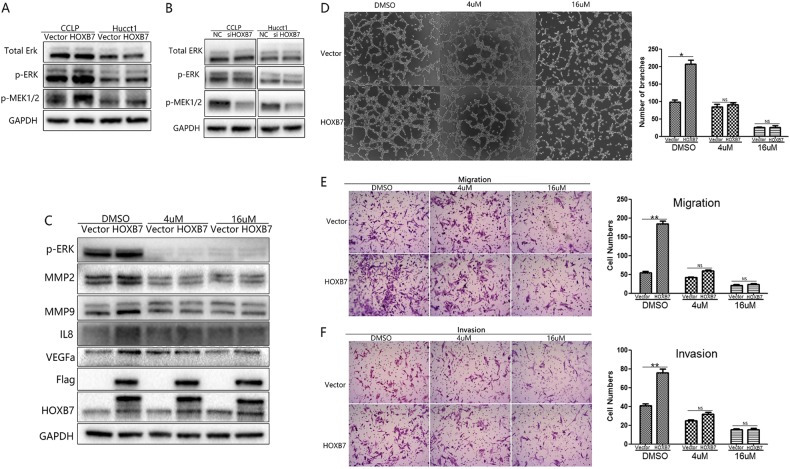
HOXB7 activates the MEK/ERK pathway to promote ICC metastasis and angiogenesis. **a**, **b** The expression levels of ERK1/2, p-ERK1/2, and p-MEK1/2 in ICC cells were analyzed by western blotting. **c** Pharmacological inhibition of ERK1/2 depressed the effect of HOXB7 on MMP2, MMP9, IL8, and VEGFa expression. **d** Pharmacological inhibition of ERK1/2 depressed the effect of HOXB7 on capillary tube formation. **e**, **f** Pharmacological inhibition of ERK1/2 depressed the effect of HOXB7 on migration and invasion.*P < 0.05, **P < 0.01, NS no significance

The results above indicated that dysregulation of HOXB7 is closely related to the malignant progression of ICC, especially with regard to metastasis and angiogenesis. However, the exact mechanism by which HOXB7 promotes ICC tumorigenesis remains unclear. As the ERK1/2 pathway is closely involved in both these functions, we next measured ERK1/2 signaling activity. In line with our expectations, we observed that p-MEK1/2 and p-ERK1/2 were upregulated in the HOXB7-overexpressing group (Fig. 6a) but that knockdown of HOXB7 downregulated p-MEK1/2 and p-ERK1/2 (Fig. 6b). To further verify our hypothesis, we used the p-ERK1/2 inhibitor SCH772984 (HY-50846, MCE, USA) to pharmacologically block the ERK1/2 pathway and investigated the angiogenesis, migration and invasion of ICC cells. Consistent with our predictions, treatment with SCH772984 ameliorated the increased expression of MMP2, MMP9, VEGF and IL8, all of which were modulated by HOXB7 (Fig. 6c). Further experiments showed that treatment with SCH772984 blocked the effects of HOXB7 on capillary tube formation (Fig. 6d) as well as migration and invasion (Fig. 6e, f). Overall, our data indicate that the ERK pathway is the primary mechanism by which HOXB7 regulates ICC cell tumorigenesis.

## Discussion

**Fig. 7:**
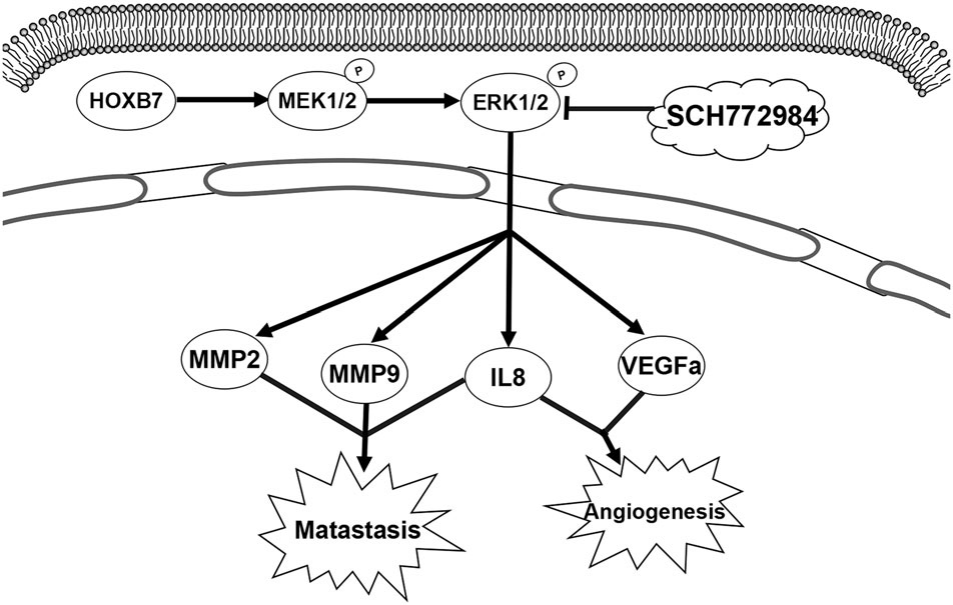
A proposed working model of how HOXB7 acts on the ERK pathway in ICC

Tumorigenesis is a complicated process with numerous aberrantly expressed genes. Among these genes, the Homeobox family has been reported to be dysregulated in a substantial number of studies. The homeodomain transcription factor HOXB7, a member of the Hox family, is closely involved in the development of cancer, and it has been reported to be aberrantly expressed in a variety of cancers, including melanoma [9], breast cancer [10–15], gastric cancer [16, 17], liver cancer [18, 19], colorectal cancer [20], and esophageal cancer [21]. However, there are few reports on the relationship between HOXB7 and ICC. In this study, we investigated the biological function of HOXB7 in ICC cell lines. HOXB7 is highly expressed in ICC cell lines, and the biological effects of HOXB7 overexpression in ICC cells are opposite to those of HOXB7 knocked-down. These results illustrate that HOXB7 plays an important role in ICC cells, and further biological function experiments involving knockdown and overexpression of HOXB7 in ICC cells confirmed the reliability of our results. We showed that upregulated HOXB7 activates the ERK pathway to upregulate the expression of MMP2, MMP9, IL8 and VEGFa, which influence ICC metastasis and angiogenesis (Fig. 7). Angiogenesis may be critical in promoting ICC tumor growth in vivo.

HOXB7 is commonly believed to be dysregulated in several types of cancers and associated with cancer progression [24]. HOXB7 upregulation has been demonstrated in colorectal cancer and esophageal squamous cell carcinoma, and HOXB7 has been shown to indicate poor prognosis in individuals with both these diseases [20, 21]. Recently, upregulated HOXB7 has been demonstrated to be a significant independent risk factor for hepatocellular carcinoma recurrence and survival after curative resection [36]. However, the expression and function of HOXB7 in ICC have remained unclear to date. Our study confirmed that HOXB7 was upregulated in ICC patients and was associated with poor prognosis in ICC.

A number of investigations has shown that HOXB7 plays an important role in modulating cell invasion and migration [17, 18]. Our results revealed that HOXB7 also had a substantial impact on the invasion and migration of ICC cells and promotes lung metastasis in vivo. Moreover, our data revealed that HOXB7 promotes ICC metastasis by upregulating MMP2, MMP9 and IL-8 expression. As is commonly known, MMPs belong to a zinc-dependent family of endopeptidases and participate in various pathological processes such as inflammatory, vascular and auto-immune disorders as well as carcinogenesis [37]. MMPs present in the extracellular matrix (ECM) have been implicated in the acquisition of migratory characteristics and result in tumor cells that are more readily able to invade surrounding tissues and metastasize to secondary sites [38]. Increased activation of MMP2 and MMP9 is mediated by the levels of IL-8, which can promote the invasive capability of a tumor. Hence, IL-8 levels have been associated with metastatic invasiveness [39]. These discoveries are consistent with our results.

HOXB7 has been reported to inhibit transgenic HER-2/Neu-induced mouse mammary tumor onset but promotes progression and lung metastasis of breast cancer [14]. However, HOXB7 also has been reported to promote tumor progression in several other cancers [16, 18, 20, 21, 36] as well as angiogenesis [9, 26]. Our results revealed that HOXB7 promotes ICC proliferation only in vivo, which may be caused by angiogenesis, one of the earliest processes involved in tumor growth and progression. Studies have also demonstrated that MMPs are involved in this progression. In an angiogenic model of tumor progression, MMP2 was shown to play an important role in the development of an angiogenic phenotype. MMP9 has also been demonstrated as a regulator of angiogenesis in a pancreatic tumor model [38]. Angiogenesis in tumors is widely believed to be regulated by a tightly controlled balance of pro- and anti-angiogenic molecules. MMP2 and MMP9 are involved in angiogenesis by disrupting basal lamina molecules to remodel the ECM during angiogenesis [40]. Moreover, angiogenic mitogens such as VEGFa can stimulate the production of MMPs in capillary endothelial cells [37], which is consistent with our results. IL8 has been reported to be a pro-angiogenic molecule [41, 42], which is consistent with the observed upregulation of IL8 in response to HOXB7 overexpression, and it has been reported that IL8 is closely associated with tumorigenesis via its effects on angiogenesis [43]. All these findings suggest that MMP2, MMP9, VEGF, and IL8 are critical during HOXB7-promoted angiogenesis, which in turn supports tumor growth and metastasis.

Our results also revealed that HOXB7 overexpression activates the ERK signaling pathway. We are the first to report that HOXB7 can activate p-MEK1/2, which is an upstream molecule of p-ERK. It has been reported that ERK can induce VEGF transcription [44] and regulate IL8 [45], MMP2, and MMP9 expression [46]. Additionally, the ERK inhibitor SCH772984, which pharmacologically blocks the ERK pathway, reduced expression of MMP2, MMP9, IL8, and VEGFa. Our results were consistent with the literature and prompted us to speculate that HOXB7 activates the ERK pathway to upregulate IL8, VEGF, MMP2 and MMP9 expression to promote ICC angiogenesis and metastasis.

In conclusion, our study confirmed that HOXB7 upregulation correlates significantly with poor prognosis in ICC. Moreover, our findings shed light on the mechanisms by which HOXB7 promotes angiogenesis and metastasis in ICC, i.e., ERK pathway activation regulates expression of MMP2, MMP9, IL8, and VEGFa. Therefore, HOXB7 expression might be a valuable prognostic indicator for ICC patients.

## Electronic supplementary material

Supplement Table 1

APC Payment Form

Laboratory Investigation
