# Supplementary material for: Upregulated expression of HOXB7 in intrahepatic cholangiocarcinoma is associated with tumor cell metastasis and poor prognosis
Source: Lab Invest. 2019 Jan 21;99(6):736–48. doi: 10.1038/s41374-018-0150-4 (PMC6760572; doi:10.1038/s41374-018-0150-4)
Supplement: Supplementary file 1 — Supplement Table 1 [file 41374_2018_150_MOESM1_ESM.docx]

| Supplement Table 1. MVD evaluated by CD31 and CD34 staining in ICC patients | | | | | |
| --- | --- | --- | --- | --- | --- |
| HOXB7 expression | n | MVD CD31  (mean ± SD) | P-value | MVD CD34  (mean ± SD) | P-value |
| High | 17 | 16.06±8.785 | ＜0.05 | 21.35±13.55 | ＜0.01 |
| Low | 11 | 8.273±4.585 |  | 7.818±5.793 |  |
